# Supplementary figures and images for: Effect of periodontal treatment on the glomerular filtration rate, reduction of inflammatory markers and mortality in patients with chronic kidney disease: A systematic review
Source: PLoS One. 2021 Jan 22;16(1):e0245619. doi: 10.1371/journal.pone.0245619 (PMC7822280; doi:10.1371/journal.pone.0245619)

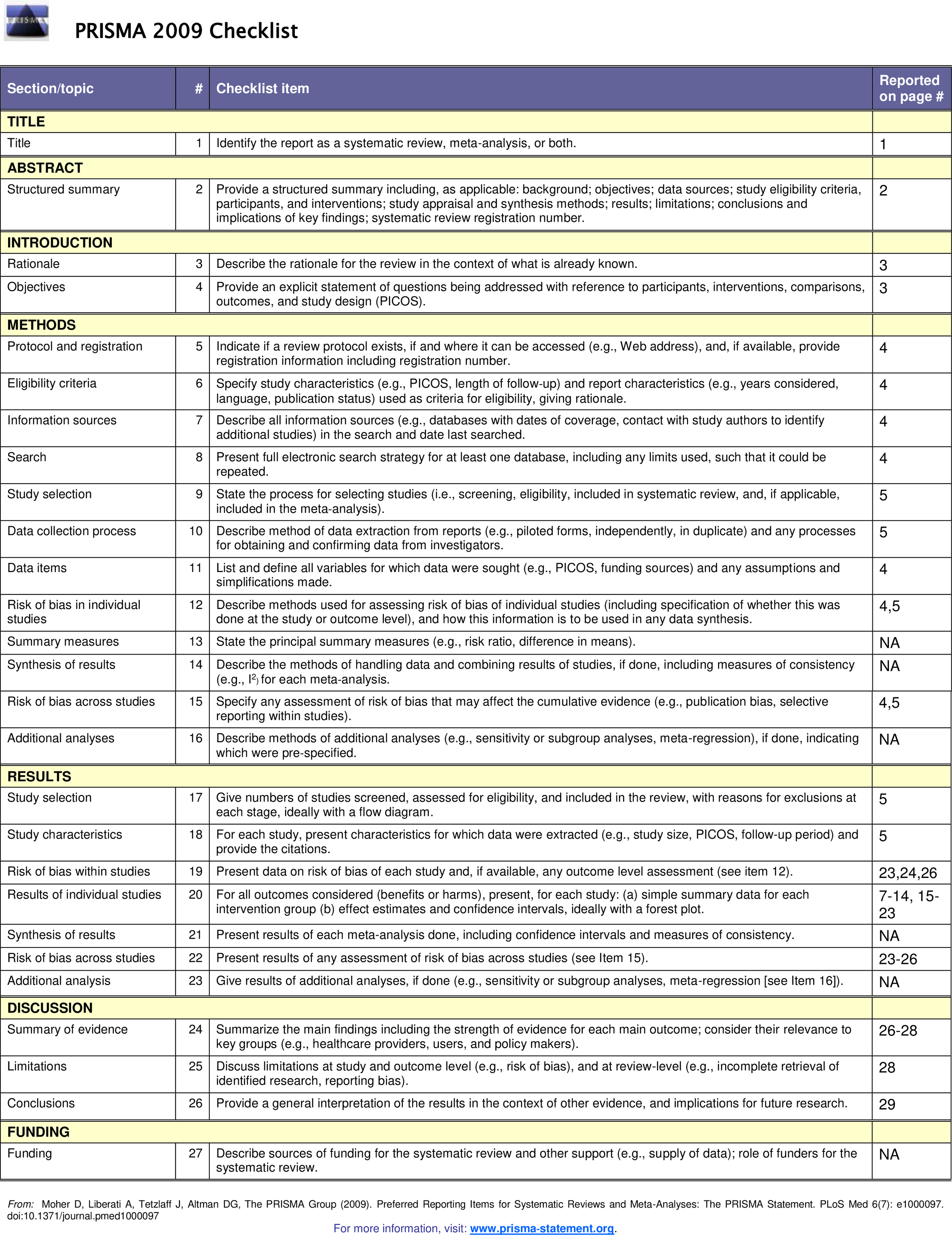

Supplement: S1 Checklist — (TIF) [file pone.0245619.s001.tif]
