# Supplementary material for: Effect of periodontal treatment on the glomerular filtration rate, reduction of inflammatory markers and mortality in patients with chronic kidney disease: A systematic review
Source: PLoS One. 2021 Jan 22;16(1):e0245619. doi: 10.1371/journal.pone.0245619 (PMC7822280; doi:10.1371/journal.pone.0245619)
Supplement: Attachment — Submitted filename: PLOS ONE REVIEW.docx [file pone.0245619.s002.docx]

Dear Editor,

Below are my contributions to the review of the article PONE-D-20-20992 titled Effect of periodontal treatment on the glomerular filtration rate, reduction of inflammatory markers and mortality in patients with chronic kidney disease: a systematic review.

Thank you for the opportunity

Kind regards

Soroye MO

**Introduction**

Line 52: kindly change i.e. to for example (Because there are other associations between periodontitis and systemic diseases)

Line 58: prevention of what? (Suggestion: prevention of periodontal disease

Line 63: kindly remove ‘Through thus’(Suggestion using clinical parameters)

**Materials and methods**

**Study selection**

Line 68-72: Any article where participants had other systemic diseases already associated with periodontitis like D/M, CVD, etc should have been **excluded** from this study

**Search Strategy**

LINES 80-83: What did you do with the articles that were duplicated since there was a likelihood of that as 2 different search engines were used (PUBMED and Web of Science)?

This should be reported here as well as under study selection

**Data collection**

Line 88 /89: kindly delete risk of, if, found, involved. Kindly replace with ‘only each risk of bias……………………….in the first instance; **where** consensus could not be reached, a third reviewer was **consulted**’

**Results**

**Study design**

Line 126: How sure were the authors of study 25 that the result they got was not due to the periodontal effect of diabetes since a 2-way relationship had been established between periodontitis and diabetes mellitus? Shouldn’t this article have been discarded in order to avoid bias? Kindly state this as a limitation to the study

Line 142: Assessment on renal disease and systemic inflammation: Kindly replace with ‘assessment **of** ………….

Periodontal assessment criteria and periodontal treatment efficacy evaluation

Line 171: kindly delete ‘for’

172: kindly replace ‘has been’ with ‘was’

267: priori………………priority
