# Supplementary material for: Effect of periodontal treatment on the glomerular filtration rate, reduction of inflammatory markers and mortality in patients with chronic kidney disease: A systematic review
Source: PLoS One. 2021 Jan 22;16(1):e0245619. doi: 10.1371/journal.pone.0245619 (PMC7822280; doi:10.1371/journal.pone.0245619)
Supplement: Attachment — Submitted filename: Response to Reviewers .docx [file pone.0245619.s003.docx]

**Response to Reviewers**

**Journal Requirements**

**1. Please ensure that your manuscript meets PLOS ONE's style requirements, including those for file naming.**

The style of the manuscript has been checked and modified as requested.

**2. Please ensure that the risk of bias for all the included studies (not just the RCTs) is assessed using a validated tool. For example, by using the Cochrane collaboration’s tool for randomized trials and the Newcastle-Ottawa Scale for nonrandomised studies**

The Newcastle-Ottawa Scale has been used (Table 5) to assess the quality of the other included studies (with control groups).

Line 91 (Material and methods section), the following sentence has been added " The quality of each non-randomized study (including at least one control group) was evaluated using the Newcastle-Ottawa Scale [22]. This scale includes 3 categorical criteria with a maximum score of 9 points: the selection of the study groups; the comparability of the groups; and the ascertainment of the outcome of interest. The quality of each study was rated using the following scoring algorithms: ≥7 points was considered as “high”, 4 to 6 points was considered as “moderate”, and ≤3 point was considered as “poor” quality study."

Line 199 (Results section), the following paragraph " Among the 14 prospective studies, 7 had no control group [24,27,30-32,34,37] and only 3 were RCTs [26,28,33] and could be used for the Cochrane collaboration’s tool for assessing risk of bias in randomized trials. Two studies showed high risk of bias concerned blinding conditions (Table 4) [26,33]. " has been replaced by " Among the 14 prospective studies, 3 were RCTs [27,29,34], 4 included at least one control group [26,30,36,37] and 7 had no control group [25,28,31-33,35,38].

The 3 RCTs could be used for the Cochrane collaboration’s tool for assessing risk of bias in randomized trials. Two studies showed high risk of bias concerned blinding conditions (Table 4) [27,34].

According to the Newcastle-Ottawa Scale, the quality of the 4 prospective studies including at least one control group and of the 4 retrospective studies were considered as moderate quality [26,30,37] and the others as high quality [36, 39-42] (Table 5).

The 7 prospective studies having no control group were considered at high risk of bias."

This modification has led to the addition of a new reference; the numbers of the other references have been modified accordingly.

**3. Please ensure that references are cited in order. For example, reference 12 doesn’t appear to be cited until the discussion.**

The references have been checked. Reference 12 had been erroneously removed from the introduction section. This has been corrected.

**4. Please include captions for your Supporting Information files at the end of your manuscript, and update any in-text citations to match accordingly.**

The caption for our Supporting Information file (S1 Checklist. Prisma 2009 checklist) has been included at the end of our manuscript. The Prisma Checklist has been modified (column "Reported on page #") in accordance with the present changes of the manuscript (revised version).

**Reviewer's Comments**

**Reviewer #1**

This manuscript is a well-written review that summarize well the knowledge on CKD and periodontal treatment. It is an up to date work. I have only minor comments.

Minor:

- l40: into consideration

- l48: bacterial

- l63: through thus??

Thank you. As suggested, modifications have been done.

**Reviewer #2**

The manuscript is good and the data support the conclusions.

The right protocol was used

The statistical analysis was performed appropriately

Article will contribute to knowledge

Minor revision are required

**Reviewer #3**

This is very interesting article. Really well written and I could not find fault in the approach taken and written to an excellent standard. The link between chronic kidney disease and periodontal disease is one that needs to be investigated further. This article rightly draws attention to the lack of carefully designed randomised clinical trials. Something that my research group are well aware of. Conclusions were valid, this is certainly an approach that should be adopted in the future when treating renal patients that periodontal treatment is provided.

**Reviewer Comments submitted as an attachment file**

**Introduction**

Line 52: kindly change i.e. to for example (Because there are other associations between periodontitis and systemic diseases)

Line 58: prevention of what? (Suggestion: prevention of periodontal disease

Line 63: kindly remove ‘Through thus’(Suggestion using clinical parameters)

Thank you for the suggestions. Modifications have been made accordingly.

**Materials and methods**

**Study selection**

Line 68-72: Any article where participants had other systemic diseases already associated with periodontitis like D/M, CVD, etc should have been **excluded** from this study

We agree that these other systemic diseases need to be taken into account. As indicated in our discussion, further methodologically robust RCTs are needed. Nevertheless, CKD patients often suffer from co-morbidities, especially ESRD patients. We have chosen, in this review, to keep all the studies in order to take into account this clinical aspect, and to help practitioners in the management of their CKD patients.

**Search Strategy**

LINES 80-83: What did you do with the articles that were duplicated since there was a likelihood of that as 2 different search engines were used (PUBMED and Web of Science)?

This should be reported here as well as under study selection

The duplicates are mentioned in the results section (in the paragraph Study selection) and in the flow diagram (Fig 1) as advised by following the PRISMA guidelines.

**Data collection**

Line 88 /89: kindly delete risk of, if, found, involved. Kindly replace with ‘only each risk of bias……………………….in the first instance; **where** consensus could not be reached, a third reviewer was **consulted**’

The sentence has been modified as suggested.

**Results**

**Study design**

Line 126: How sure were the authors of study 25 that the result they got was not due to the periodontal effect of diabetes since a 2-way relationship had been established between periodontitis and diabetes mellitus? Shouldn’t this article have been discarded in order to avoid bias? Kindly state this as a limitation to the study

In the study 25 *(study 25 has become study 26 after the addition of a reference)*, the experimental group (Chronic Kidney Disease + Diabetes + Periodontitis) was compared to 2 control groups, one including patients with Diabetes + Periodontitis and one including patients with Periodontitis only, taking into consideration the relationship between periodontitis and diabetes mellitus. Following the Journal requirements,  the quality of this study has now been assessed using the Newcastle-Ottawa scale.

Line 142: Assessment on renal disease and systemic inflammation: Kindly replace with ‘assessment of ………….

Periodontal assessment criteria and periodontal treatment efficacy evaluation

Line 171: kindly delete ‘for’

172: kindly replace ‘has been’ with ‘was’

267: priori………………priority

Thank you for the suggestions. Modifications have been made accordingly.

Line 267 (line 282 in the revised version), "a priori" has been written in italics to avoid confusion
